# Budget Impact Analysis of Fixed Dose Versus Weight-Based Dosing Regimen of Nivolumab and Pembrolizumab in the Treatment of Non-Small Cell Lung Cancer

**DOI:** 10.3390/vaccines8040730

**Published:** 2020-12-03

**Authors:** Sanjana Monirul, Marthe Rigal, Kader Chouahnia, Mélisande Le Jouan, Maxime Apparuit, Adrien Paix, Anne Jacolot, Laurent Zelek, Boris Duchemann

**Affiliations:** 1 Department of Pharmacy, Hôpital Avicenne, Hôpitaux Universitaires Paris Seine Saint-Denis, 93000 Bobigny, France; sanjana.monirul@aphp.fr (S.M.); marthe.rigal@aphp.fr (M.R.); maxime.apparuit@aphp.fr (M.A.); anne.jacolot@aphp.fr (A.J.); 2 Department of Medical and Thoracic Oncology, Hôpitaux Universitaires Paris Seine Saint-Denis, 93000 Bobigny, France; kader.chouahnia@aphp.fr (K.C.); laurent.zelek@aphp.fr (L.Z.); 3 OMEDIT Ile-De-France, 75014 Paris, France; melisande.le-jouan@aphp.fr; 4 Institut de Radiothérapie de Bobigny, rue Lautréamont, 93000 Bobigny, France; adrienpaix@gmail.com; 5 Gustave Roussy Cancer Campus, Laboratoire D’immunomonitoring en Oncologie, CNRS-UMS 3655 and INSERM-US23, 94805 Villejuif, France

**Keywords:** budget impact analysis, lung cancer, nivolumab, pembrolizumab, fixed dose, cancer immunotherapy

## Abstract

In 2018, dosing regimens of the two most prescribed immune check point inhibitors (ICI), nivolumab (Opdivo^®^) and pembrolizumab (Keytruda^®^), in the treatment of lung cancer were changed from weight-based dosing to fixed dosing. The aim of this study was to compare the economic impact of this change in our university hospital group and then across Ile-de-France, the most inhabited French region. A budget impact analysis (BIA) has been performed on the French public health insurance data. The duration of treatment and the weight of the patients were calculated using data from the patients treated at our health facility and from clinical studies. The cost of treatment was calculated at the local level of our health facility and then for Ile-de-France. Our model demonstrates an additional cost of €550,115 in our hospital and €9,704,778 in Ile-de-France for a fixed dose prescription in 2018. In 2019, the BIA concluded an additional cost, according to the respective low and high assumptions, of €556,969 and €756,544 locally and € 10,201,027 to €14,486,141 for Ile-de-France for an equivalent efficacy between the two different drug dosing regimens of nivolumab and pembrolizumab. The adoption of the fixed dose regimen would lead, according to the least expensive hypothesis, to an additional cost of 26% for the ICI. These results encourage reflection on the strict adoption of this dosage modification. The option of maintaining the free choice between a prescription adapted to weight or in a fixed dose seems a relevant option and should be considered.

## 1. Introduction

Lung cancer has been the most common cancer in the world for several decades, with 1.8 million cases diagnosed in 2012. It is also the deadliest, with 1.6 million deaths in the same year [1].

In France, the number of new cases diagnosed in 2017 was estimated at 49,109 (32,260 in men and 16,849 in women) with a median age of diagnosis of 66 years in men and 65 years in women (2015 data) [2]. Lung cancer is the leading cause of cancer death in France, all populations combined, with 30,991 deaths estimated in 2017 with a median age at the time of death of 68 years for men and 67 years for women. It is also the first cause of death among all causes in men between 45 and 64 years old and the second cause of cancer death in women after breast cancer [3].

Advanced non-small-cell lung carcinoma (NSCLC) treatment has been based on cytotoxic chemotherapy for decades, with a doublet of platinum in combination with several others agents [4]. The first therapeutic evolution was related to the discovery of targetable oncogenic mutation using mainly a small tyrosine kinase inhibitor. Nevertheless, this situation of tumor with oncogenic addiction remains the exception [5].

The main revolution of the last decade has been the discovery of immune checkpoint inhibitors (ICI), the first being the Programmed Death-ligand 1 (PD(L)1)inhibitor. These immunotherapies are efficient and well tolerated in NSCLC. Despite being imperfect biomarkers, they achieved a better response rate than chemotherapy in second line with unprecedented duration of response and improved overall survival (OS), representing a breakthrough in the standard of care. ICI have become the backbone of treatment for advanced NSCLC [6]. In 2016 and 2017, two anti-PD-1s (programmed cell death 1), nivolumab and pembrolizumab, were approved as the new 2nd-line standards for the treatment of metastatic non-small-cell lung carcinoma (NSCLC). Pembrolizumab is also a first-line option if the tumor expresses PD-L1 (Programmed death-ligand 1) ≥50%. PD-L1 immunohistochemistry represents the most valuable and robust predictive biomarker for ICI response in clinical practice. Indeed, response rates (RR) and outcome in terms of OS increase with higher PD-L1 expression [7,8,9].

More recently, pembrolizumab received approval in France when combined with platinum-based chemotherapy in patients regardless of PD-L1 expression [10,11].

### 1.1. Economic Impact

The financial mass committed in Ile-de-France for the treatment by immunotherapy of lung cancer amounts to tens of millions of euros, which weighs on health facilities. Anticancer drugs represent an increasingly important expense item. Thus, the INCa (French National Cancer Institute) estimated that in 2014, 55.7% of the total 2.87 billion euros of expenditure was dedicated to anticancer drugs [12]. Based on data from the ATIH (Technical Agency for Information on Hospital Care), we estimated the expenditure related to nivolumab and pembrolizumab at around 100 million euros in 2018 in Ile-de-France, all indications combined, private and public healthcare facilities combined, and regardless of the administrative status of the ICI (temporary authorization for use = ATU or regulatory approval by the French Health Agency or European Health Agency = AMM). As the time required to obtain the regulatory approval (AMM) is generally long, there is a temporary authorization for use (ATU) system in France. As long as the efficacy and the safety have been established by clinical studies, this system lets a potentially innovative drug in serious pathologies be available to patients more quickly, before obtaining AMM. This device exclusively concerns treatments for which the first trials have yielded very promising data. It is issued by the ANSM (French Agency for the Safety of Health Products) for drugs intended to treat serious or rare diseases for which there is no appropriate treatment or the implementation of the treatment cannot be postponed. Medicines under ATU are covered 100% by the French public health insurance.

**Figure 1:**
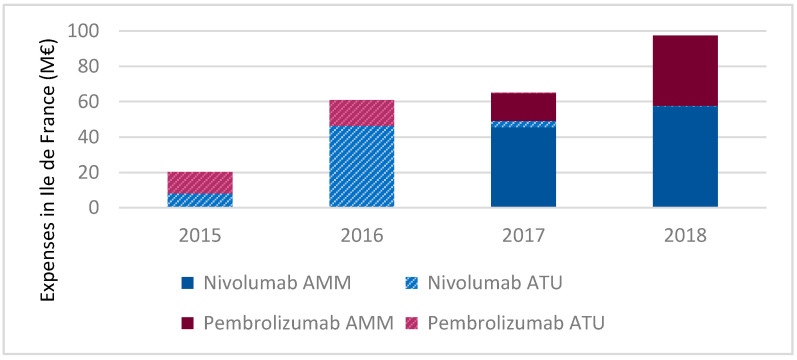
Evolution of the amount of anti-PD-1 spending in public and private healthcare facilities in Ile-de-France from January 2015 to December 2018 (data processed after extraction from the ScanSanté platform that allows users to make queries in order to obtain formatted results, processed by ATIH).

The evolution of expenditure of nivolumab and pembrolizumab between 2015 and 2018 is detailed in Figure 1.

After dazzling and continuous growth in cost observed since its launch (+476% for nivolumab between 2015 and 2016), the progression of nivolumab observed a slowdown with only a slight increase in 2017 (+6.1%) and in 2018 (+17.2%). However, for pembrolizumab, after a slowdown in 2017, we again noted an increase in 2018 (+149%) probably linked to an extension of its indications (locally advanced or metastatic urothelial carcinoma, metastatic non-squamous NCSLC, recurrent or metastatic head and neck squamous cell carcinoma, and Stage III melanoma with lymph node involvement).

While expenses committed for ICI already weight on overall drug spending, the dosage regimen was changed in 2018 from a weight-adapted dose to a fixed dose, raising fears of a further increase in spending.

### 1.2. New Dosage Strategy

Without renewing clinical studies comparing directly the efficacy and safety of these dosage regimens, marketing authorization has been changed for nivolumab and pembrolizumab from a dosage adapted to weight to a fixed dosage by the FDA [13] first and then followed by Canadian and European authorities. This was conducted on the unique basis of pharmacological and pharmacokinetic studies [14,15] carried out by the laboratory marketing these drugs.

Nivolumab was reduced from an approved dose (from first randomized studies) of 3 mg/kg every 2 weeks to a fixed dose of 240 mg every two weeks in NSCLC indication [16,17]. Concerning pembrolizumab, the dose of 2 mg/kg every three weeks was changed to a fixed dose of 200 mg at the same frequency [18].

It is in this context that a team from Avicenne Hospital wanted to measure the financial impact of the implementation of this new dose regimen in the treatment of lung cancer, by comparing the cost of prescribing nivolumab and pembrolizumab as a fixed dose compared to the cost of the treatment at the reference of weight-based dosing. The study was carried out first at our hospital level and then at the regional level of Ile de France. This study was conducted before the approval of ICI in combination to chemotherapy in the first line setting and before the result of atezolizumab in the second line setting of advanced NSCLC [19,20].

## 2. Materials and Methods

### 2.1. Methodological Overview

The economic impact was measured by evaluating the additional cost generated by the use of a fixed-dose compared to a weight-based dose. A budget impact analysis (BIA) was carried out, according to the recommendations of the HAS (Haute Autorité de Santé, Paris, France) [21], for the year 2018, at the Avicenne Hospital and then at the Ile de France region level [22] to measure the financial impact of the new dosing strategy for nivolumab and pembrolizumab. A projection was then carried out over a time horizon of one year (2019). Ile-de-France is the densest and most populated French region with 12,174,880 inhabitants in 2017 representing 19% of the population of metropolitan France [23]. Avicenne Hospital located in Ile-de-France is the only University Tertiary Hospital of Seine-Saint-Denis department serving a population of 1,623,111 inhabitants in 2017 [24].

This analysis was performed from the perspective of payers in France, i.e., French public health insurance.

### 2.2. Database

PMSI (i.e., program for medicalization information systems) data extraction was performed using the shared and mutualized decision-making tool called DIAMANT (Décisionnel Inter-Ars pour la Maîtrise et l’ANTicipation) [25].

Patients included were those who received one of the two drugs to treat a metastatic NSCLC, whether in hospital or in a day case chemotherapy unit. PMSI data for Avicenne Hospital were compared with data from our prescription software CHIMIO^®^ in order to check the consistency of data. Data were collected from 1 January 2018 to 31 December 2018. This study covers all type of healthcare facilities located in Ile-de-France having medicine, surgery, and obstetrics activity, regardless of the tarification applied.

The expenditure data for nivolumab and pembrolizumab were taken from the ScanSanté platform [26]. From these data, the following were calculated:

- the number of patients treated with each drug according to the treatment line,
- the number of cures received per year per patient,
- the average number of cures received per year by all patients treated.

The data extracted from the CHIMIO^®^ software were verified for each patient and drug administration, using the establishment’s information systems (ORBIS^®^ patient file, Ar-Kdos^®^ RCP files).

### 2.3. Time Horizon

A time horizon was set to integrate all the medical care parameters and expected results. The BIA was initially carried out for the year 2018 in our care facility, and then it was projected over a time horizon of one year, the year 2019. This choice seems justified regarding the constant evolution of therapeutic strategies in the treatment of lung cancer.

### 2.4. Target Population

The target population of the study is defined as the population likely to be impacted by the intervention studied over the defined time horizon; these are all patients treated for metastatic 1st line NSCLC or 2nd line NSCLC depending on the molecule studied.

There are thus two types of populations treated:

▪ in 1st line, patients treated with pembrolizumab as monotherapy indicated for metastatic NSCLC in adults whose tumors express PD-L1 with a ≥ 50% tumor proportion score (TPS), with no EGFR or ALK positive tumor mutations.
▪ in 2nd line, patients included were:
  ◦ patients treated with nivolumab, as monotherapy for the treatment of locally advanced or metastatic NSCLC, without scoring condition,
  ◦ patients treated with pembrolizumab as monotherapy for the treatment of locally advanced or metastatic NSCLC in adults whose tumors express PD-L1 with a TPS greater than or equal to 1%.

An estimate of the population was calculated from PMSI data by selecting the active file of patients treated in 2018 with nivolumab and/or pembrolizumab in Avicenne Hospital then in Ile-de-France for a “Malignant tumor of the bronchi and lung” (code C34 of the International Classification of Diseases, ICD-10 or CIM-10 in France). This calculation of the analyzed population was completed with the data entered in CHIMIO^®^. For this purpose, patients treated with nivolumab and pembrolizumab in 2018 at Avicenne Hospital were extracted according to the treatment line to assess prescription habits. For pembrolizumab, the distribution rate observed between 1st and 2nd line in 2018 in Avicenne Hospital was applied to the population of Ile-de-France.

Among patients with NSCLC (85% of patients with lung cancer [27]), only those with advanced and/or metastatic stages are eligible, which represent, respectively, 30 and 40% of NSCLC. In addition, around 40% of patients with disease managed at the localized stage (surgery or radio-chemotherapy) will develop metastatic disease at distance. Therefore, 82% of patients will be likely to receive treatment at the metastatic stage. Without EGFR or ALK tumor mutations, found in about 13.5% of cases, systemic chemotherapy treatment is the standard:

▪ In the first line, due to an altered general condition, an advanced age of the patients or the presence of comorbidities, the HAS, in its opinion, considers that around 80% of patients will receive treatment. In addition, in the first line, the number of patients likely to benefit from pembrolizumab is low due to tumor overexpression of PD-L1 ≥ 50%. Among the patients included in the pivotal Keynote 024 study [28], approximately 25% had a PD-L1 status ≥ 50%. More conservatively, in this study, we assumed that 20% of patients have a PD-L1 status ≥ 50%. Those 20% patients are then potentially affected by a prescription for pembrolizumab.
▪ In the 2nd line, in its opinion, the HAS estimates that among the patients treated in the first line, only 40% of patients will be eligible for a new treatment. However, with regard to treatment with ICI, which is theoretically better tolerated than treatment with chemotherapy, it is assumed that 50% of patients will be eligible for second-line treatment. In addition, we assume that patients who received first-line treatment with ICI will not be eligible for a new immunotherapy treatment even if there are no data on the prescription of ICI in the 2nd line. Finally, among the patients still to be treated in the 2nd line, the number of patients likely to benefit from nivolumab and/or pembrolizumab is estimated at 100% due to the fact that nivolumab prescription does not depend on PD-L1 status.

**Figure 2:**
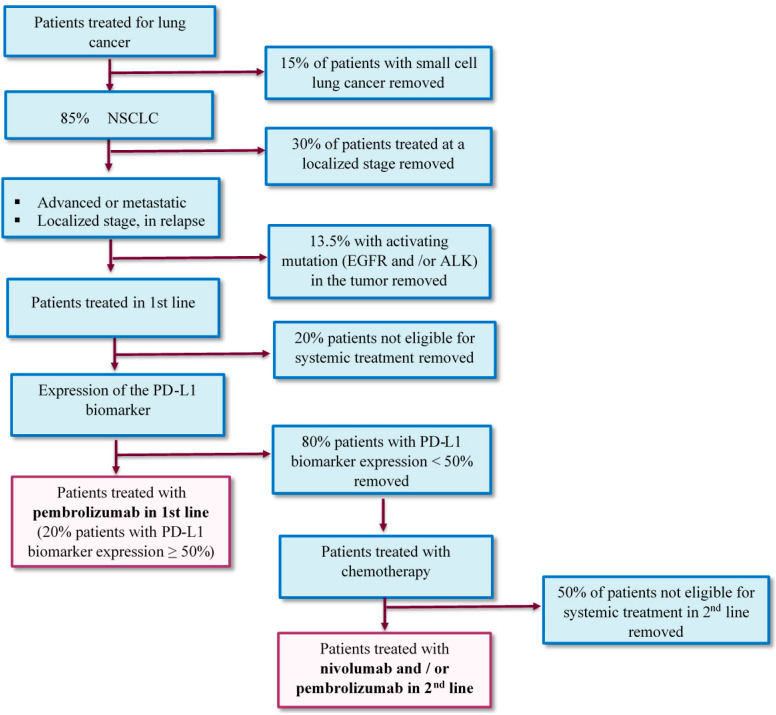
Target patient population in the base case analysis. **ICI used are in bold**.

Figure 2 illustrates how the target population in 1st and 2nd line of treatment was estimated for our study.

### 2.5. Evolution of the Analysis Population between 2018 and 2019

In general terms, as part of a BIA, a growth or decrease rate of the target population must be applied in order to take into account the evolution of the incidence of lung cancer over the reporting period. Regarding lung cancer, an annual growth of 1.5% of the active file of patients with metastatic NSCLC was estimated by taking the average of the annual growth rates of the population hospitalized in Ile-de-France and having been coded in “C34-T.M. BRONCHES AND LUNG” in PMSI data. The data extracted concern the period from January 2012 to December 2018 (Table 1).

### 2.6. Cost of Nivolumab and Pembrolizumab

The cost of the drug was set according to the “tarif de responsabilité” (financial base used by French Health Insurance to calculate its reimbursement) on 1 January 2019. The price per mg used in the study was €10.58 for nivolumab [29] and €26.84 for pembrolizumab [30] (all taxes included). Costs related to hospitalization were not taken into account in this study because they are considered equivalent for the two strategies.

### 2.7. Patients’ Weight

The average weight of a patient treated for NSCLC was estimated from the cohort of patients followed and treated by nivolumab or pembrolizumab at Avicenne Hospital for this indication. We recorded and analyzed the weight of all patients (*n* = 139) as prescribed in CHIMIO^®^ from 2015 to 2018. It was fixed at 68 kg (95% Confidence Interval (CI): 66.3–69.7).

### 2.8. Duration of Treatment

The duration of treatment was estimated from the average number of cycles received per year by patients monitored and treated in Avicenne Hospital with ICI (since August 2015 for nivolumab). Patients treated with nivolumab in the 2nd metastatic line in our hospital received an average of 8.5 cycles per year at the frequency of one cycle every two weeks. As pembrolizumab was available at the end of 2017, the decline in median patient follow-up is too short to calculate the average number of cycles and may not take long responders into account. Data from the pivotal studies KEYNOTE 024 [28] in the 1st line and KEYNOTE 010 [31] in the 2nd line were used. The average number of cycles of pembrolizumab received was 10.5 cycles and 4.9 cycles, respectively, at the frequency of one cycle every three weeks.

### 2.9. Scenarios

**Figure 3:**
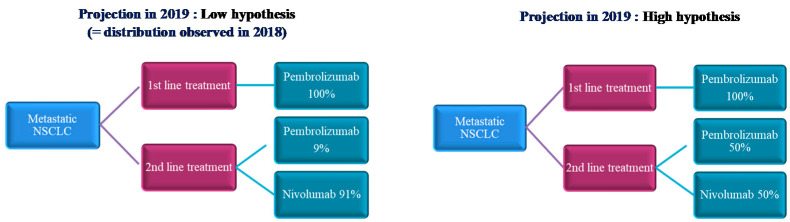
Illustration of the budget impact model over the time horizon.

The cost of a treatment over one year for each drug was calculated according to two scenarios: fixed dose and weight-based dose. For each scenario, two working hypotheses (low and high hypothesis) allow us to take into account the distribution of prescriptions between nivolumab and pembrolizumab in the 2nd line treatment, as both can be used. The additional cost is defined as the cost difference between the fixed dose scenario and the weight-based dose. The additional cost is finally calculated for each molecule studied and per treatment line according to the two working hypotheses. For 2018, we use the strictly observed distribution between the prescriptions of pembrolizumab and nivolumab in the second line in Avicenne Hospital (i.e., 9% market share for pembrolizumab and 91% for nivolumab). For 2019, two different market share assumptions are made for the population treated in the 2nd line. The first hypothesis or “low hypothesis” consists of keeping the same distribution as that observed in 2018: no change in prescribing practices. This low assumption of second-line pembrolizumab prescriptions is justified because on the one hand nivolumab was marketed earlier in the second-line, and on the other hand, its prescription does not rely on testing for PD-L1 status. The second hypothesis or “high hypothesis” provides for a completely homogeneous distribution of prescriptions between nivolumab and pembrolizumab in the 2nd line. This high hypothesis of pembrolizumab prescription distribution is made because of a frequency of administration every three weeks. The different hypotheses used are illustrated in Figure 3.

### 2.10. Sensitivity Analyses

**Figure 4:**
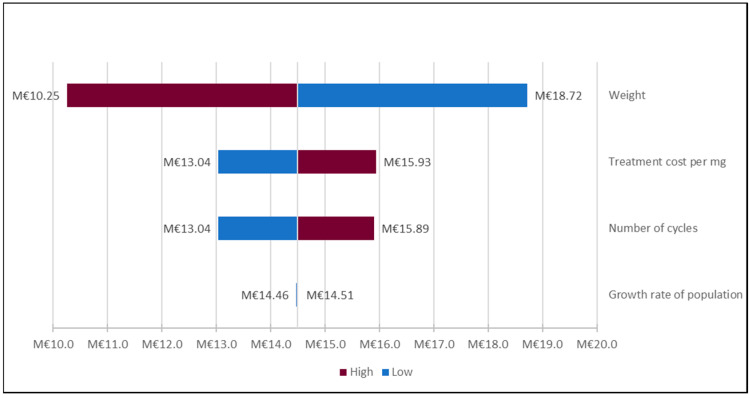
Tornado chart: univariate deterministic sensitivity analysis comparing the influence of different parameters on cost-savings.

A sensitivity analysis was performed to assess the parameters for which the range had the greatest impact on cost savings. We used the Tornado chart as the graphical representation of the univariate deterministic sensitivity analysis. A Tornado graph is a specific form of a histogram that allows us to compare the uncertainty for each parameter by varying the values of each parameter in turn, the other parameters remaining at their value retained in the reference analysis [32]. The sensitivity analysis was performed with all base care parameters: weight, treatment cost, number of cycles, and growth rate of the population. For all parameters we created a range by applying +/− 10% to all base values. The Tornado chart is available in Figure 4. We used cost data in Ile-de-France for the high hypothesis in 2019 to create the diagram.

## 3. Results

The target population was calculated by taking into account the number of patients treated in Avicenne Hospital and Ile-de-France in 2018 (PMSI data). A projection after application of a population change rate allows the calculation of the target population for 2019 (Table 2). This change was set from the growth rate of the population treated in this indication in Ile-de-France. An average of the annual growth rates of the population hospitalized in Ile-de-France for the indication C34 “Malignant neoplasm of bronchus and lung” allowed the estimation of this annual change rate at + 1.5% in this indication.

The estimated cost of treatment per year was set at €56,364 for pembrolizumab prescribed at a fixed dose and at €38,325 in the case of a dose proportional to weight (Table 3).

The estimated cost for the second line treatment was evaluated at €21,582 for a fixed dose and €18,343 for a weight-based dose for nivolumab and €26,303 and €17,885 for pembrolizumab. The calculations are detailed in Table 4.

The annual additional cost per patient and per molecule was calculated according to two scenarios (fixed dose and weight-based dose) from the price per mg of the drug, the average weight of patients extracted from CHIMIO^®^ estimated at 68 kg, and the annual treatment duration; estimated at 8.5 cycles for the 2nd line for nivolumab (CHIMIO^®^ data) and at 10.5 cycles for the 1st line and 4.8 cycles for the 2nd line for pembrolizumab, according to data from the Keynote 024 [28] and Keynote 010 [31] studies. The calculations are detailed in Table 5.

The global annual additional cost per molecule was then calculated for the entire analyzed population (Table 6). For 2019, the cost of the 2nd line treatment was also calculated according to two market share assumptions, since the two drugs are available: a low hypothesis taking into account the distribution observed in our establishment between the prescriptions of nivolumab and pembrolizumab and a high hypothesis with an equivalent distribution between the two drugs.

The BIA generated, for a fixed-dose prescription in 2018, an additional cost of €550,115 in our health facility and €9,704,778 in Ile-de-France. In 2019, the BIA concluded an additional cost, according to the low and high assumptions, from €556,969 to €756,544 locally and from €10,201,027 to €14,486,141 for Ile-de-France (Table 7). Indeed, for each hypothesis studied, the adoption of the fixed dose resulted in an increase in expenditure of +47% for pembrolizumab and of +18% for nivolumab.

**Figure 5:**
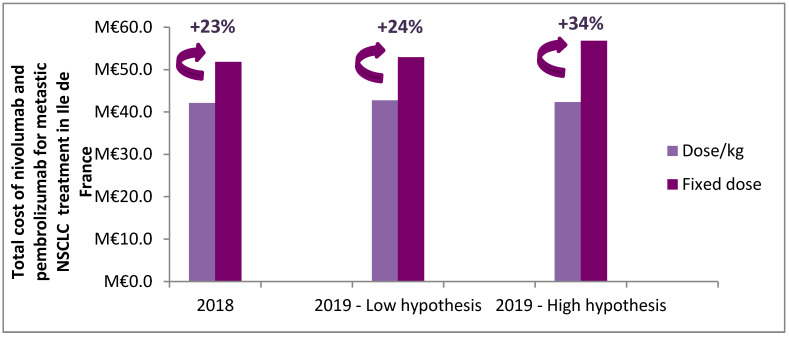
Estimate of the extra cost of switching to the fixed dose compared to the weight-based dose in Ile-de-France.

The adoption of the fixed dose in 2019 would increase ICI’s expenditure for all lines by 34% under the high hypothesis and by 24% with the low hypothesis for the Ile-de-France as illustrated in Figure 5.

In the Tornado chart (Figure 4), the length of each bar represents the importance of the sensitivity of cost-savings to changes in the parameters included in the analysis. The representation of the diagram is such that the influence of the parameters is ranked in descending order: the most influential parameter being at the top and the least influential being at the bottom. The color of the bars corresponds to the level of the parameter of interest (in purple, the high value of the parameter, in blue, the low value of the parameter) compared to its reference value (median). The univariate sensitivity analyses demonstrate that the parameters with the greatest potential impact on cost-savings are patient’s weight, treatment cost per mg, and duration of treatment with nivolumab and pembrolizumab (number of cycles). Indeed, by varying the average weight by 10%, cost-saving varies between M€10.25 to M€18.72. The most conservative estimate of savings is M€10.25 when the average weight is at the upper end of the range (74.8 kg).

## 4. Discussion

The arrival of immunotherapy and promising results in terms of prolonged patient survival raise fears of increased health costs. The strategical disruption observed with the ICI is linked to economic concerns for our health system. In this context, pharmacoeconomic analysis is a major health decision-making tool regarding the financing and use of health interventions which allows a more efficient allocation of ever scarcer resources. While the use of international clinical data is generally the rule for drug use approval, epidemiological data vary from country to country and economic data are not always transferable from one country to another.

At Avicenne Hospital, at the time of the dosage change of nivolumab in April 2018, we observed over the first four months of 2018 that for 77% of the prescriptions of nivolumab for a NSCLC, prescribed doses were lower than the fixed dose of 240 mg. Conversely, 17% had a higher dose than the fixed dose and only 6% of prescriptions would not be changed, discrepancies varying from +30% to +84% between prescribed dose and fixed dose. This observation was the starting point for questions about the financial impact of this decision.

Indeed, the additional cost generated at the level of a hospital and at the regional level by the modification of the strategy is major. It is expected, based on our assessment, to increase the annual expenditure of pembrolizumab and nivolumab by approximately 25%.

In our economic evaluation, the equivalence of the clinical results with the two dosage strategies compared is implied. The first pivotal studies of pembrolizumab did not demonstrate any clinically significant exposure-effectiveness relationship for pembrolizumab doses from 2 to 10 mg/kg but mainly demonstrated equivalent efficacy regardless of these doses [31,33,34]. Since the 200 mg single dose falls within this range, it seems that efficacy is equivalent regardless of the dose. The first clinical trials and models suggest that the exposure-response relationship reaches a plateau beyond 2 mg/kg [23], without statistically significant improvement in the efficacy of doses from 2 to 10 mg/kg [21]. Furthermore, an analysis including Keynote 001, 002, 006, 010, and 024 showed that the pharmacokinetics of pembrolizumab were equivalent for the fixed dose and the weight-based dose with a uniform exposure-response relationship [35]. These results were confirmed in randomized clinical trials without demonstrating any statistically significant difference in results (response rate, progression-free survival: PFS and overall survival: OS) between the two dose options (2 mg/kg versus 10 mg/kg) in melanoma and NSCLC (Keynote 002 [36], Keynote 010 [21], and Keynote 001 [37]. Similar results are observed for nivolumab. Indeed, from a pharmacological point of view, nivolumab has a broad therapeutic index and is well tolerated up to doses of 10 mg/kg [38]. Phase I studies demonstrated that dose increases ranging from 0.3 to 10 mg/kg had only a marginal influence on anti-PD-1 receptor occupancy on the surface of T cells [39]. Moreover, Feng Y et al., in 2017, published a meta-analysis evaluating the relationship between levels of exposure to nivolumab and OS as well as adverse effects based on the results of four different studies of squamous cell NSCLC and non-squamous cell [40]. The analyses show no significant difference in OS and adverse effects between the doses received (1, 3 or 10 mg/kg) for each of the histologies of NSCLC. The authors concluded that nivolumab has a relatively flat dose-response curve between 1 and 10 mg/kg every two weeks, which in their point of view, supports the use of the initially approved dose of 3 mg/kg every two weeks for patients with metastatic NSCLC. The change in dosage strategy has not been supported by any new clinical study but has been validated from pharmacokinetic studies only.

This change in dosage proposed by the manufacturer quickly raised a number of questions. Indeed, if we consider the average weight of the French population, 77 kg for men and 63 kg for women in 2003, according to INSEE (French National Institute of Statistics and Economic Studies) [41], we can easily imagine that the use of a dose of 200 mg of pembrolizumab for all patients is an unnecessarily high dose. A dose adapted to the appropriate weight for an average French adult would be 154 mg for men and 126 mg for women. For an American patient, this dose would also be too high with an average weight of 82 kg in the United States [42]. Therefore, uncertainties remain regarding a possible increase in toxicities related to the increase in doses received. These toxicities must be reported to the competent authorities (pharmacovigilance).

The medico-economic aspect is essential to ensure the sustainability of the French health care system and to continue to guarantee access to optimal care for cancer patients. Therefore, the change in dosage was accompanied by a number of arguments in favor of a cost offset. The use of an identical dose for all treated patients allows us to form the hypothesis of a possible reallocation of preparations in case of treatment deprogramming.

Regarding patients’ weight, to be closer to reality, it was considered relevant to integrate the real life data of a cohort of patients rather than a theoretical weight.

Furthermore, only the cost of the drug was taken into account in our evaluation because we hypothesized that changing dosing strategies had no impact on preparation and/or administration costs. In addition, the cost of drugs taken into account for our study is based on the financial base (or “tarif de responsabilité”) as determined centrally by the administration, while the actual prices are very often negotiated between the buyer (i.e., CEPS or French medicine pricing committee) and the manufacturer. However, as these negotiated prices are confidential, they could not be included in the evaluation. This again creates the risk of overestimating the economic impact which is dependent on negotiations and therefore on the health facility. In this regard, in 2017, Norum et al. [43], in their BIA on the introduction of pembrolizumab in the second-line treatment of NSCLC in northern Norway, strongly criticize this principle of price confidentiality, which makes it difficult to carry out and publish transparent pharmaco-economic analyses on new drugs around the world.

Finally, theoretical benefits of the fixed drug dosage regime were not analyzed in our study, such as a reduction in prescription errors (dosage error), a simplification of preparation and therefore improved working conditions for pharmaceutical staff, a reduction in the waste of remaining medication, a reduction in the risk of infection (by reducing the number of manipulations in the same treatment vial), a reduction in the loss of medication (remainder), a reduction in patient waiting times, and a reduction in the risk of administration errors [14,15].

### Comparison with the Literature

In Canada, the Institut national d’excellence en santé et en services sociaux (INESS), which carries out health economic evaluations for drug reimbursement decisions, in its notice to the Québec’s Minister of Health and Social services concerning the use of nivolumab in NSCLC published in August 2016, estimates $374 M (average of $124.7 M/year) as the amount of additional costs that could be added to the budget of the health facility during the first three years following the addition of a recognized indication for nivolumab. These estimates are based on the assumption that 6329 patients with an average weight of 70 kg would be treated during these years with an average cycle number of 13.74 cycles (with 2184 patients in the first year) [44].

In September 2018, the Canadian Programme de Gestion Thérapeutique des Médicaments (PGTM) published two evaluation reports of new dosing strategies: weight-based dosing, fixed dosing or weight-based dose limited by a maximum dose for nivolumab [45] and pembrolizumab [46]. With a solid literature review, PGTM found that there are no clinical studies that have compared the different dosing strategies for nivolumab with each other or that aim to demonstrate equivalence of efficacy or safety. However, considering pharmacokinetic studies and pharmacological properties, the PGTM considers that it would be acceptable to administer nivolumab at a rate of 3 mg/kg up to a maximum of 240 mg every two weeks for all patients and all indications. By extrapolation, it is also considered reasonable a weight dose of 6 mg/kg up to a maximum of 480 mg every four weeks. The pharmaco-economic evaluation carried out by the PGTM shows that the average weight in the university hospitals of Quebec (72.5 kg) is 10% lower than the weight used to determine the fixed dose (80 kg) and for a price of $4260 per dose every two weeks, the switch to the fixed dose has an economic impact amounting to millions of euros (increase in nivolumab expenditure by 10%). It also estimates that the recommendations of the PGTM (i.e., 3 mg/kg up to a maximum dose of 240 mg every two weeks or 6 mg/kg up to a maximum dose of 480 mg every four weeks) would make it possible to generate 10% savings.

Moreover, the PGTM proposes different hypotheses to reduce the cost of using nivolumab. To test these hypotheses, it uses budget impact data estimated by the INESS (gross impact over three years). To limit losses, the following strategies are proposed:

▪ For the largest centers, regroup patients receiving nivolumab visits over a few targeted days each week in order to be able to use the rest of each vial for the next patient.
▪ In centers with a smaller volume of patients, the use of dose standardization (dose-banding) up to a maximum dose is proposed. In the PGTM model, the standard doses are rounded to the nearest 20 mg, unless this exceeds 5% of the difference from the dose he or she would have received based on his or her weight (3 mg/kg), in which case, they increase to the higher dose (rounded up to the next 20 mg), up to a maximum of 240 mg.

Using this model, the PGTM measured the impact that four different dosing strategies could have: use of the dose according to the weight of 3 mg/kg, fixed-dose of 240 mg, weight-based dose of 3 mg/kg up to a maximum dose of 240 mg and weight-based dose (3 mg/kg) up to 240 mg with dose standardization (possible doses: 120, 140, 180, 200, 220, and 240 mg). According to this model, the use of the fixed dose is the most expensive strategy. The use of the dose by weight with a maximum dose is the strategy which allowed the greatest savings, around 12 to 15%, while the use of a dose according to weight allowed savings of the order of from 8 to 10%. The use of standardized doses would mean savings of around 10 to 14%.

It has to be noted that Canada has approved the fixed dose only as an alternative option to the weight-based dose.

In August 2017, INESS performed a BIA for pembrolizumab on the use of pembrolizumab in 1st line NSCLC expressing PD-L1 ≥ 50% [47]. INESS estimates, over three years, additional costs of $87.8 M (average of $29.3 M/year). These estimates are based on the assumption that 1079 patients would be treated during these years (annual average number of 360 patients).

Some similar studies have been conducted to date.

In 2017, Goldstein et al. [48] performed a BIA from the payer’s point of view in the United States according to the recommendations of the International Society in which they compared the economic impact of the use of pembrolizumab at a fixed dose of 200 mg versus a dose adapted to the weight (2 mg/kg) in the 1st line treatment of metastatic NSCLC. The authors calculated the target population size and weight of patients who could be treated with pembrolizumab annually and estimated the number of cycles to receive based on the PFS and OS results from Keynote 024 and a Weibull extrapolation model. They showed that the use of the weight-based dose versus the fixed dose would allow an annual saving of 24% on the expenses of pembrolizumab or approximately 826 million dollars for the United States without impacting the results.

As with nivolumab, in the evaluation report for pembrolizumab, the PGTM notes that there are no published studies aimed at demonstrating equivalence or a difference in efficacy or safety between the different dosage strategies. However, based on the evidence from currently published pharmacokinetic studies and pharmacological properties, it is believed that the dose of pembrolizumab of 2 mg/kg would be comparable to the fixed dose of 200 mg every three weeks. Therefore, the PGTM considers that it would be acceptable to give 2 mg/kg up to a maximum of 200 mg every three weeks. Pharmaco-economic evaluation conducted by the PGTM shows that using the 2 mg/kg dose for an average weight of 72.5 kg, at an estimated price of $6380 per dose every three weeks (or approximately $8505 per 28 day period), represents a financial burden of millions of dollars, which weighs heavily on the budgets of the health facility in Quebec. It also finds that the weight of 100 kg, used to determine the fixed dose is approximately 30% higher than the average weight of patients treated in the university hospitals of Quebec. Administration of the fixed dose of 200 mg to all patients would increase spending on pembrolizumab by approximately 30%, or $8800 per dose (thus approximately $11,733 per a 28-day period). The weight dose of 2 mg/kg every three weeks up to a maximum dose of 200 mg would generate additional savings by limiting the dose for patients over 100 kg [46].

Finally, in 2019, Bayle and al. [49] performed a BIA comparing weight-based doses to fixed-doses for nivolumab and pembrolizumab, used for any type of cancer at the Gustave Roussy level for the year 2018. The study was conducted for a total of 978 perfusions (560 of nivolumab in 103 patients and 418 of pembrolizumab in 125 patients) mainly treated for lung cancer and melanoma at Gustave Roussy. Data were collected from January to April 2018. They estimated the annual extra cost at €477,120 for nivolumab and €1,613,898 for pembrolizumab. Moreover, they expected an annual budget impact of €55,162,211 at the French national level for the year 2017.

Our results are consistent with all these studies found in the literature.

## 5. Conclusions

The adoption of this new regimen (switch from a weight-based dose to a fixed dose) could lead, according to the least costly hypothesis, to an additional cost of 26% linked to an ICI prescribed for a NSCLC. These dose modifications have not been clinically validated and moreover no benefit is expected for the patients. These results encourage reflection on the strict adoption of this dosage modification and to seek a consensus with the health authorities concerned with the actions to be taken. In the absence of national recommendations, maintaining a free choice between a weight-adapted or a fixed dose prescription seems a relevant option.

## Figures and Tables

**Table 1:** Growth rates of the population treated in Ile-de-France for metastatic NSCLC.

Year	2012	2013	2014	2015	2016	2017	2018
Number of patients	6981	7036	7207	7351	7574	7449	7642
Growth rates		+0.8%	+2.4%	+2.0%	+3.0%	−1.7%	2.6%

**Table 2:** Calculation of the population of patients treated with ICI in Avicenne Hospital and Ile-de-France for lung cancer (PMSI data).

	Pembrolizumab	Nivolumab
2018 (PMSI data)		
Avicenne Hospital	20	84
Ile-de-France	261	1876
2019 (projection after application of population change rate)		
Avicenne Hospital	20 *	85 *
Ile-de-France	265 *	1904 *

* Numbers have been rounded up or down to show the whole number of patients.

**Table 3:** Annual cost calculation of treatment with pembrolizumab for patients treated in the 1st line.

Posology A	Weight (kg) B	Total (mg) C = A × B	Price (All Taxes Included)/mg D	Cost per Administration C × D	Number of Cycles E	Treatment Cost per Patient (C × D) × E
**Pembrolizumab**						
2 mg/kg	68	136	€26.84 *	€3650	10.5	€38,325
200 mg	68	200	€26.84 *	€5368	10.5	€56,364

* Financial base per mg.

**Table 4:** Annual cost calculation of treatment with nivolumab and pembrolizumab for patients treated in the 2nd line.

Posology A	Weight (kg) B	Total (mg) C = A × B	Price (All Taxes Included)/mg D	Cost per Administration C × D	Number of Cycles E	Treatment Cost per Patient (C × D) × E
**Nivolumab**						
3 mg/kg	68	204	€10.58 *	€2158	8.5	€18,343
240 mg	68	240	€10.58 *	€2539	8.5	€21,582
**Pembrolizumab**						
2 mg/kg	68	136	€26.84 *	€3650	4.9	€17,885
200 mg	68	200	€26.84 *	€5368	4.9	€26,303

* Financial base per mg.

**Table 5:** Annual additional cost per patient and per molecule of the fixed dose compared to the weight-based dose.

ICI	Nivolumab	Pembrolizumab	
		1st Line	2nd Line
Cost of Weight-based dose	€18,343	€38,325	€17,885
Cost of fixed dose	€21,582	€56,364	€26,303
Additional treatment cost per year and per patient	€3239	€18,039	€8418

**Table 6:** Global annual additional cost per molecule for the analyzed population.

Nivolumab	2018	2019	
		Low Hypothesis	High Hypothesis
Avicenne Hospital	€272,076	€277,032	€152,215
Ile-de-France	€6,076,364	€5,948,192	€3,268,238
**Pembrolizumab** **(1st line + 2nd line)**	**2018**	**2019**	
		**Low hypothesis**	**High hypothesis**
Avicenne Hospital	€278,039	€279,937	€604,329
Ile-de-France	€3,628,414	€4,252,835	€11,217,904

**Table 7:** Global annual additional cost for the two molecules for the analyzed population.

Nivolumab + Pembrolizumab	2018	2019	
		Low Hypothesis	High Hypothesis
Avicenne Hospital	€550,115	€556,969	€756,544
Ile-de-France	€9,704,778	€10,201,027	€14,486,141

## References

[1] Fact Sheets by Cancer. http://globocan.iarc.fr/Pages/fact_sheets_cancer.aspx.

[2] Le Cancer du Poumon—Les Cancers les Plus Fréquents. http://www.e-cancer.fr/Professionnels-de-sante/Les-chiffres-du-cancer-en-France/Epidemiologie-des-cancers/Les-cancers-les-plus-frequents/Cancer-du-poumon.

[3] Haute Autorité de Santé—Cancer Broncho-Pulmonaire: Le Parcours de Soins Doit Préserver en Priorité Une Qualité de Vie. https://www.has-sante.fr/portail/jcms/c_1651595/fr/cancer-broncho-pulmonaire-le-parcours-de-soins-doit-preserver-en-priorite-une-qualite-de-vie.

[4] Schiller J.H., Harrington D., Belani C.P., Langer C., Sandler A., Krook J., Zhu J., Johnson D.H. (2002). Comparison of Four Chemotherapy Regimens for Advanced Non–Small-Cell Lung Cancer. N. Engl. J. Med..

[5] Barlesi F., Mazieres J., Merlio J.-P., Debieuvre D., Mosser J., Lena H., Ouafik L., Besse B., Rouquette I., Westeel V. (2016). Routine molecular profiling of patients with advanced non-small-cell lung cancer: Results of a 1-year nationwide programme of the French Cooperative Thoracic Intergroup (IFCT). Lancet.

[6] Remon J., Passiglia F., Ahn M.-J., Barlesi F., Forde P.M., Garon E.B., Gettinger S., Goldberg S.B., Herbst R.S., Horn L. (2020). Immune Checkpoint Inhibitors in Thoracic Malignancies: Review of the Existing Evidence by an IASLC Expert Panel and Recommendations. J. Thorac. Oncol..

[7] Brahmer J., Horn L., Hossein B., Ramalingam S., Pluzanski A., Burgio M., Garassino M., Chow L., Gettinger S., Crino L. (2019). O.02 Long-term Survival Outcomes with Nivolumab (NIVO) in Pts with Previously Treated Advanced Non-Small Cell Lung Cancer (NSCLC): Impact of Early Disease Control and Response. J. Thorac. Oncol..

[8] Herbst R.S., Garon E.B., Kim D.-W., Cho B.C., Perez-Gracia J.L., Han J.-Y., Arvis C.D., Majem M., Forster M.D., Monnet I. (2020). Long-Term Outcomes and Retreatment among Patients With Previously Treated, Programmed Death-Ligand 1‒Positive, Advanced Non‒Small-Cell Lung Cancer in the KEYNOTE-010 Study. J. Clin. Oncol..

[9] Aguilar E., Ricciuti B., Gainor J., Kehl K., Kravets S., Dahlberg S., Nishino M., Sholl L., Adeni A., Subegdjo S. (2019). Outcomes to first-line pembrolizumab in patients with non-small-cell lung cancer and very high PD-L1 expression. Ann. Oncol..

[10] Gandhi L., Rodríguez-Abreu D., Gadgeel S., Esteban E., Felip E., De Angelis F., Domine M., Clingan P., Hochmair M.J., Powell S.F. (2018). Pembrolizumab plus Chemotherapy in Metastatic Non–Small-Cell Lung Cancer. N. Engl. J. Med..

[11] Paz-Ares L., Luft A., Vicente D., Tafreshi A., Gümüş M., Mazières J., Hermes B., Çay Şenler F., Csőszi T., Fülöp A. (2018). Pembrolizumab plus Chemotherapy for Squamous Non–Small-Cell Lung Cancer. N. Engl. J. Med..

[12] Institut National du Cancer, Le Prix des Médicaments Anticancéreux, Jun. https://www.e-cancer.fr/Expertises-et-publications/Catalogue-des-publications/Le-prix-des-medicaments-anticancereux.

[13] FDA, Approved Drugs—Modification of the Dosage Regimen for Nivolumab. https://www.fda.gov/drugs/resources-information-approved-drugs/modification-dosage-regimen-nivolumab.

[14] Zhao X., Suryawanshi S., Hruska M., Feng Y., Wang X., Shen J., Vezina H.E., McHenry M.B., Waxman I.M., Achanta A. (2017). Assessment of nivolumab benefit–risk profile of a 240-mg flat dose relative to a 3-mg/kg dosing regimen in patients with advanced tumors. Ann. Oncol..

[15] Freshwater T., Kondic A., Ahamadi M., Li C.H., De Greef R., De Alwis D., Stone J.A. (2017). Evaluation of dosing strategy for pembrolizumab for oncology indications. J. Immunother. Cancer.

[16] Brahmer J.R., Reckamp K.L., Baas P., Crinò L., Eberhardt W.E., Poddubskaya E., Antonia S., Pluzanski A., Vokes E.E., Holgado E. (2015). Nivolumab versus Docetaxel in Advanced Squamous-Cell Non–Small-Cell Lung Cancer. N. Engl. J. Med..

[17] Borghaei H., Paz-Ares L., Horn L., Spigel D.R., Steins M., Ready N.E., Chow L.Q., Vokes E.E., Felip E., Holgado E. (2015). Nivolumab versus Docetaxel in Advanced Nonsquamous Non–Small-Cell Lung Cancer. N. Engl. J. Med..

[18] Robert C., Ribas A., Wolchok J.D., Hodi F.S., Hamid O., Kefford R., Weber J.S., Joshua A.M., Hwu W.-J., Gangadhar T.C. (2014). Anti-programmed-death-receptor-1 treatment with pembrolizumab in ipilimumab-refractory advanced melanoma: A randomised dose-comparison cohort of a phase 1 trial. Lancet.

[19] Rittmeyer A., Barlesi F., Waterkamp D., Park K., Ciardiello F., Von Pawel J., Gadgeel S.M., Hida T., Kowalski D.M., Dols M.C. (2017). Atezolizumab versus docetaxel in patients with previously treated non-small-cell lung cancer (OAK): A phase 3, open-label, multicentre randomised controlled trial. Lancet.

[20] Fehrenbacher L., Spira A., Ballinger M., Kowanetz M., Vansteenkiste J., Mazieres J., Park K., Smith D., Artal-Cortes A., Lewanski C. (2016). Atezolizumab versus docetaxel for patients with previously treated non-small-cell lung cancer (POPLAR): A multicentre, open-label, phase 2 randomised controlled trial. Lancet.

[21] HAS Guide Méthodologique—Choix méthodologiques pour l’évaluation économique à la HAS, October. https://www.hassante.fr/portail/upload/docs/application/pdf/2011-11/guide_methodo_vf.pdf.

[22] Haute Autorité de Santé—Analyse d’Impact Budgétaire: La HAS Enrichit l’Évaluation Médico-Économique des Produits de Santé. https://www.has-sante.fr/portail/jcms/c_2747893/en/analyse-d-impact-budgetaire-la-has-enrichit-l-evaluation-medico-economique-des-produits-de-sante.

[23] Population Légale de l’Île-de-FRANCE—Insee Flash Ile-de-France. https://www.insee.fr/fr/statistiques/4270719.

[24] Dossier Complet—Département de la Seine-Saint-Denis (93) | Insee. https://www.insee.fr/fr/statistiques/2011101?geo=DEP-93.

[25] Keyrus, Les Agences Régionales de Santé se Dotent d’un Outil de Pilotage Mutualisé à la Pointe de Diamant. http://keyrus-prod.s3.amazonaws.com/uploads/media/SuccessStories_ARS-2012.pdf.

[26] ScanSanté—Accès Aux Données Facilité | ATIH. https://www.atih.sante.fr/actualites/scansante-acces-aux-donnees-facilite.

[27] HAS Tumeur Maligne, Affection Maligne du Tissu Lymphatique ou Hématopoïétique Mélanome Cutané, January. https://www.has-sante.fr/upload/docs/application/pdf/2012-03/ald_30_guide_melanome_web.pdf.

[28] Reck M., Rodríguez-Abreu D., Robinson A.G., Hui R., Csőszi T., Fülöp A., Gottfried M., Peled N., Tafreshi A., Cuffe S. (2016). Pembrolizumab versus Chemotherapy for PD-L1–Positive Non–Small-Cell Lung Cancer. N. Engl. J. Med..

[29] Assurance Maladie OPDIVO, Base des Médicaments et Informations Tarifaires (BDM_IT). http://www.codage.ext.cnamts.fr/codif/bdm_it//fiche/index_lis_ucd.php?p_code_cip=&p_nom_commercial=OPDIVO&p_nb=2&p_site=AMELI&p_homol_retro=&p_homol_taa=taa.

[30] Assurance Maladie KEYTRUDA, Base des Médicaments et Informations Tarifaires (BDM_IT). http://www.codage.ext.cnamts.fr/codif/bdm_it//fiche/index_lis_ucd.php?p_code_cip=&p_nom_commercial=KEYTRUDA&p_nb=2&p_site=AMELI&p_homol_retro=&p_homol_taa=taa.

[31] Herbst R.S., Baas P., Kim D.-W., Felip E., Pérez-Gracia J.L., Han J.-Y., Molina J., Kim J.-H., Arvis C.D., Ahn M.-J. (2016). Pembrolizumab versus docetaxel for previously treated, PD-L1-positive, advanced non-small-cell lung cancer (KEYNOTE-010): A randomised controlled trial. Lancet.

[32] Morelle M., Plantier M., Dervaux B., Pagès A., Deniès F., Havet N., Perrier L. (2018). Méthodes d’analyse et de traitement des données de coût : Approches par « micro-costing » et « gross-costing ». Revue d’Épidémiologie et de Santé Publique.

[33] Patnaik A., Kang S.P., Rasco D., Papadopoulos K.P., Elassaiss-Schaap J., Beeram M., Drengler R., Chen C., Smith L., Espino G. (2015). Phase I Study of Pembrolizumab (MK-3475; Anti-PD-1 Monoclonal Antibody) in Patients with Advanced Solid Tumors. Clin. Cancer Res..

[34] Garon E.B., Rizvi N.A., Hui R., Leighl N., Balmanoukian A.S., Eder J.P., Patnaik A., Aggarwal C., Gubens M., Horn L. (2015). Pembrolizumab for the Treatment of Non–Small-Cell Lung Cancer. N. Engl. J. Med..

[35] Garon E., Reck M., Rodriguez-Abreu D., Robinson A., Hui R., Tibor C., Fulop A., Gottfried M., Peled N., Tafreshi A. Use of a 200-mg fixed dose of pembrolizumab for the treatment of advanced non–small cell lung cancer (NSCLC). Proceedings of the the 17th World Conference on Lung Cancer.

[36] Hamid O., Puzanov I., Dummer R., Schachter J., Daud A., Schadendorf D., Blank C.U., Cranmer L.D., Robert C., Pavlick A.C. (2017). Final analysis of a randomised trial comparing pembrolizumab versus investigator-choice chemotherapy for ipilimumab-refractory advanced melanoma. Eur. J. Cancer.

[37] Robert C., Ribas A., Hamid O., Daud A.I., Wolchok J.D., Joshua A.M., Hwu W.-J., Weber J.S., Gangadhar T.C., Joseph R.W. (2016). Three-year overall survival for patients with advanced melanoma treated with pembrolizumab in KEYNOTE-001. J. Clin. Oncol..

[38] Topalian S.L., Hodi F.S., Brahmer J.R., Gettinger S.N., Smith D.C., McDermott D.F., Powderly J.D., Carvajal R.D., Sosman J.A., Atkins M.B. (2012). Safety, Activity, and Immune Correlates of Anti–PD-1 Antibody in Cancer. N. Engl. J. Med..

[39] Brahmer J.R., Drake C.G., Wollner I., Powderly J.D., Picus J., Sharfman W.H., Stankevich E., Pons A., Salay T.M., McMiller T.L. (2010). Phase I Study of Single-Agent Anti-Programmed Death-1 (MDX-1106) in Refractory Solid Tumors: Safety, Clinical Activity, Pharmacodynamics, and Immunologic Correlates. J. Clin. Oncol..

[40] Feng Y., Wang X., Bajaj G., Agrawal S., Bello A., Lestini B., Finckenstein F.G., Park J.-S., Roy A. (2017). Nivolumab Exposure–Response Analyses of Efficacy and Safety in Previously Treated Squamous or Nonsquamous Non–Small Cell Lung Cancer. Clin. Cancer Res..

[41] L’obésité en France: Les Écarts Entre Catégories Sociales S’accroissent—Insee Première. https://www.insee.fr/fr/statistiques.

[42] A McDowell M., Fryar C.D., Ogden C.L. (2009). Anthropometric reference data for children and adults: United States, 1988-1994. Vital- Health Stat. Data Natl. Health Surv..

[43] Norum J., Antonsen M.A., Tollåli T., Al-Shibli K., Andersen G., Svanqvist K.H., Helbekkmo N. (2017). Pembrolizumab as second-line therapy in non-small cell lung cancer in northern Norway: Budget impact and expected gain—A model-based analysis. ESMO Open.

[44] INESS Opdivo—Cancer du Poumon Non à Petites Cellules, August. https://www.inesss.qc.ca/fileadmin/doc/INESSS/Inscription_medicaments/Avis_au_ministre/Aout_2016/Opdivo_CPNPC_2016_08.pdf.

[45] (PGTM) Programme de Gestion Thérapeutique des Médicaments NIVOLUMAB—Quelle Stratégie Posologique Devrait-on Privilégier: Dose en Fonction du Poids, Dose Fixe ou Dose en Fonction du Poids Avec Une Dose Maximale? Rapport d’Évaluation, September. http://www.pgtm.org/documentation/FSW/Nivolumab_Strat%C3%A9gie%20posologique.pdf.

[46] (PGTM) Programme de Gestion Thérapeutique des Médicaments PEMBROLIZUMAB—Quelle Stratégie Posologique Devrait-on Privilégier: Dose en Fonction du Poids, Dose Fixe ou Dose en Fonction du Poids Avec Une Dose Maximale? Rapport d’Évaluation, September. http://www.pgtm.org/documentation/FSW/Pembrolizumab_Strat%C3%A9gie%20posologique.pdf.

[47] INESS Keytruda—Cancer du Poumon Non à Petites Cellules—Avis d’Ajout d’Une Indication Reconnue à la Liste Établissements, August. https://www.inesss.qc.ca/fileadmin/doc/INESSS/Inscription_medicaments/Avis_au_ministre/Novembre_2017/Keytruda_1re_int_CPNPC_2017_08.pdf.

[48] Goldstein D.A., Gordon N., Davidescu M., Leshno M., Steuer C.E., Patel N., Stemmer S.M., Zer A. (2017). A Phamacoeconomic Analysis of Personalized Dosing vs Fixed Dosing of Pembrolizumab in Firstline PD-L1-Positive Non–Small Cell Lung Cancer. J. Natl. Cancer Inst..

[49] Bayle A., Besse B., Annereau M., Bonastre J. (2019). Switch to anti-programmed cell death protein 1 (anti-PD-1) fixed-dose regimen: What is the economic impact?. Eur. J. Cancer.

